# Differences in anterior peripheral pathologic myopia and macular pathologic myopia by age and gender

**DOI:** 10.1007/s00417-021-05217-w

**Published:** 2021-05-21

**Authors:** Cassie A. Ludwig, Nick Boucher, Namrata Saroj, Darius M. Moshfeghi

**Affiliations:** 1 Byers Eye Institute, Department of Ophthalmology, Stanford University, 2452 Watson Court, Palo Alto, CA 94303 USA; 2 Retina Service, Department of Ophthalmology, Massachusetts Eye and Ear, Harvard Medical School, Boston, MA 02114 USA; 3 Vestrum Health, 1121 S. Naper Blvd., Naperville, IL 60540 USA; 4 All Eyes Consulting, LLC, 300 East 59th Street 3401, New York, NY 10022 USA


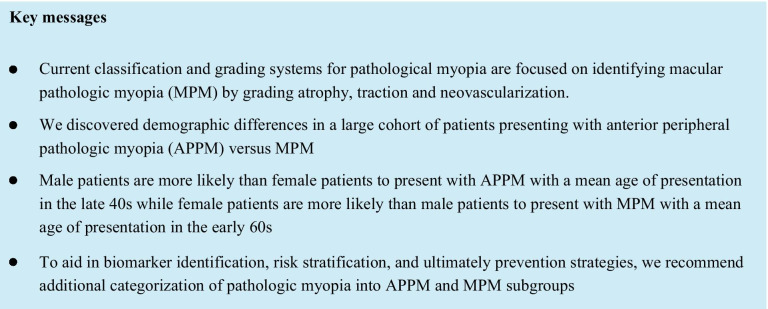


## Dear Editor,

Uncorrected refractive error is the second most common cause of blindness and moderate and severe vision impairment in the world [1]. The etiology of high myopia is multifactorial with both environment and genetics playing a role in axial elongation [2]. Most literature on high myopia focuses on posterior findings such as myopic maculopathy and posterior staphyloma [3]. However, we demonstrated in a single center that high myopia can be classified into anterior and posterior high myopia, with different risk profiles based on gender and age, suggesting different etiologies for these presentations [4]. We tested these findings in a larger dataset.

Using aggregated de-identified data from the Vestrum Health Database (Naperville, IL, USA), we performed a retrospective study of patients with high myopia enrolled from January 1, 2015 to December 31, 2019. Study participants were identified using ICD-9/ICD-10 codes 360.21/H442. Predetermined ICD diagnoses were then used to classify patients with high myopia into previously described subgroups, with slight modifications for clarity (anterior pathologic myopia, APM, renamed anterior peripheral pathologic myopia, APPM; posterior pathologic myopia, PPM, renamed macular pathologic myopia, MPM) [4]. Subgroups were defined as APPM (lattice degeneration, rhegmatogenous retinal detachment, retinal tear, retinoschisis), MPM (myopic maculopathy, macular hole, staphyloma, angioid streaks, retinal neovascularization, cystoid macular degeneration, exudative retinopathy, retinal pigmented epithelium detachment, vitreomacular adhesion, epiretinal membrane), combined pathologic myopia (CPM; diagnosis from each of the aforementioned two categories), and isolated high myopia (IHM) [4]. The influence of gender and age on classification were examined using crude bivariate analyses (chi-squared, ANOVA, relative risk). Analyses were performed based on individual eye classification. Statistical assumptions were met.

The study population included 54,875 patients (106,243 eyes, Table 1). The following subgroups were identified (percentage, mean age at classifying diagnosis): MPM (17.2%, 63.5 y) — accounting for 13.6% of those > 86 y, APPM (29.4%, 47.9 y) — accounting for 26.9% of those < 35 y, CPM (10.6%, 58.5 y), IHM (42.8%, 57.6 y). Females had a 20.9% greater risk (95% CI, 17.6 to 24.4%) of MPM as compared to males. Males had an 18.0% greater risk (95% CI, 15.8 to 20.3%) of APPM as compared to females.

**Table 1:** Demographics of patients with high myopia and pathologic myopia by anterior and posterior subtypes

Characteristic	Total population (N = 1,117,133)	APPM (N = 383,726)	MPM (N = 608,541)	CPM (N = 73,748)	IHM (N = 51,118)	*p* value
Age, *mean (SD)*	55.8 (17.2)	47.9 (16.9)	63.5 (14.2)	58.5 (13.6)	57.6 (17.3)	< .001
Age Groups, *n (%)*						
< = 35	15,338 (14.4)	8,251 (26.4)	804 (4.4)	780 (6.9)	5,503 (12.1)	< .001
36–45	11,350 (10.7)	4,803 (15.4)	1,063 (5.8)	873 (7.7)	4,611 (10.1)	< .001
46–55	19,889 (18.7)	6,699 (21.4)	2,527 (13.8)	2,283 (20.2)	8,380 (18.4)	< .001
56–65	27,969 (26.3)	6,920 (22.1)	5,356 (29.3)	3,924 (34.8)	11,769 (25.9)	< .001
66–75	19,571 (18.4)	3,351 (10.7)	5,068 (27.8)	2,485 (22.0)	8,667 (19.1)	< .001
76–85	8,737 (8.2)	932 (3.0)	2,527 (13.8)	751 (6.7)	4,527 (10.0)	< .001
> = 86	3,191 (3.0)	286 (0.9)	871 (4.8)	178 (1.6)	1,856 (4.1)	< .001
Gender, *n (%)*						
Male	40,039 (37.7)	13,015 (41.6)	6,088 (33.3)	4,798 (42.5)	16,138 (35.5)	< .001
Female	66,204 (62.3)	18,235 (58.4)	12,173 (66.7)	6,479 (57.5)	29,317 (64.5)	< .001

*SD*, standard deviation; *APPM*, anterior peripheral pathologic myopia; *MPM*, macular pathologic myopia; *CPM*, combined pathologic myopia; *IHM*, isolated high myopia

These results align with previous findings of unique subgroups of high myopia with differences in age and gender: (1) MPM patients are more likely to be older females and (2) APPM patients to be younger males, and (3) younger patients are more likely to be diagnosed with APPM while (4) older patients are more likely to have MPM. Differences between males and females are likely driven by a known increased risk of RRD in males [5].

This study is strengthened by its large number of participants. The study was limited by its reliance on ICD coding and lack of refraction data; more myopic refractive error is linked to CPM and MPM as compared to APPM [4].

Herein, we again demonstrate the disparity in populations affected by APPM and MPM based on age and gender. The current ATN grading and classification system does not account for anterior retinal pathology as a disease-defining entity [3]. As anterior retinal pathology is both common and potentially visually significant, it is important to supplement the current system with that suggested herein when classifying and risk-stratifying patients with high myopia, as well as searching for genetic etiologies.

## References

[1] Bourne RRA, Stevens GA, White RA (2013). Causes of vision loss worldwide, 1990–2010: a systematic analysis. Lancet Glob Health.

[2] Chakraborty R, Read SA, Vincent SJ, Ang M, Wong TY (2020). Understanding Myopia: Pathogenesis and Mechanisms. Updates on Myopia: A Clinical Perspective.

[3] Ruiz-Medrano J, Flores-Moreno I, Ohno-Matsui K (2020). Validation of the recently developed ATN classification and grading system for myopic maculopathy. Retina.

[4] Ludwig CA, Shields RA, Chen TA (2018). A novel classification of high myopia into anterior and posterior pathologic subtypes. Graefes Arch Clin Exp Ophthalmol.

[5] Sheu S-J, Ger L-P, Chen J-F (2007). Male sex as a risk factor for pseudophakic retinal detachment after cataract extraction in Taiwanese adults. Ophthalmology.

