# Correction to: Differences in anterior peripheral pathologic myopia and macular pathologic myopia by age and gender

**DOI:** 10.1007/s00417-023-06053-w

**Published:** 2023-04-05

**Authors:** Cassie A. Ludwig, Nick Boucher, Namrata Saroj, Darius M. Moshfeghi

**Affiliations:** 1 Byers Eye Institute, Department of Ophthalmology, Stanford, University, 2452 Watson Court, Palo Alto, CA 94303 USA; 2 Retina Service, Department of Ophthalmology, Massachusetts Eye and Ear, Harvard Medical School, Boston, MA 02114 USA; 3 Vestrum Health, 1121 S. Naper Blvd., Naperville, IL 60540 USA; 4 All Eyes Consulting, LLC, 300 East 59Th Street 3401, New York, NY 10022 USA

**Correction to: Graefe’s Archive for Clinical and Experimental Ophthalmology (2021) 259:3511–3513**

10.1007/s00417-021-05217-w

We revised Table 1 after a change in methodology prior to submission, but erroneously left the table headers of Table 1 from the first data run. The remainder of the data within the manuscript and table are accurate.

This erratum would like to correct the column headers of Table 1 as:

Total population (N = 106, 243), APPM (N = 31,250), MPM (N = 18,261), CPM (N = 11,277), IHM (N = 45,455).

We apologize for this error.

